# Antimicrobial Biosynthetic Potential and Phylogenetic Analysis of Culturable Bacteria Associated with the Sponge *Ophlitaspongia* sp. from the Yellow Sea, China

**DOI:** 10.3390/md20100588

**Published:** 2022-09-21

**Authors:** Lei Chen, Xue-Ning Wang, Hong-Yu Bi, Guang-Yu Wang

**Affiliations:** 1 Department of Bioengineering, School of Marine Science and Technology, Harbin Institute of Technology at Weihai, Weihai 264209, China; 2 Shanghai Center for Systems Biomedicine, Key Laboratory of Systems Biomedicine (Ministry of Education), Shanghai Jiao Tong University, Shanghai 200240, China

**Keywords:** actinobacteria, antimicrobial activity, marine bacteria, diversity, marine natural products, sponge, PKS, NRPS

## Abstract

Sponge-derived bacteria are considered to be a promising source of novel drugs, owing to their abundant secondary metabolites that have diverse biological activities. In this study, we explored the antimicrobial biosynthetic potential and phylogenetics of culturable bacteria associated with the sponge *Ophlitaspongia* sp. from the Yellow Sea, China. Using culture-dependent methods, we obtained 151 bacterial strains, which were then analysed for their antimicrobial activities against seven indicator strains. The results indicate that 94 (62.3%) of the 151 isolated strains exhibited antimicrobial activities and inhibited at least one of the indicator strains. Fifty-two strains were selected for further phylogenetic analysis using 16S rRNA gene sequencing, as well as for the presence of polyketide synthase (PKS) and non-ribosomal peptide synthetase (NRPS) genes. These 52 strains belonged to 20 genera from 18 families in 4 phyla, including *Actinobacteria*, *Bacteroidetes*, *Firmicutes*, and *Proteobacteria*. Five strains with PKS genes and ten strains with NRPS genes were detected. Among them, two strains contained both PKS and NRPS genes. *Notoacmeibacter* sp. strain HMA008 (class *Alphaproteobacteria*) exhibited potent antimicrobial activity; thus, whole genome sequencing methods were used to analyse its secondary metabolite biosynthetic gene clusters. The genome of HMA008 contained 12 biosynthetic gene clusters that potentially encode secondary metabolites belonging to compound classes such as non-ribosomal peptides, prodigiosin, terpene, β-lactones, and siderophore, among others. This study indicates that the sponge *Ophlitaspongia* sp. harbours diverse bacterial strains with antimicrobial properties and may serve as a potential source of bioactive compounds.

## 1. Introduction

Marine ecological environments contain numerous symbiotic relationships established between microorganisms and a wide range of eukaryotic hosts [1]. Marine sponges are multi-cellular filter-feeding invertebrates that harbour various bacteria, representing approximately 41 known phyla [2,3]. These sponge-derived bacteria are responsible for a series of functional metabolic activities, including antibacterial, antifungal, anti-tumour, and anti-infective properties, and have been recognised as a promising source of pharmaceutically bioactive substances [4,5]. More than 5300 different metabolites have been isolated from sponges and their associated microorganisms, some of which are currently in use in clinical trials [6]. Both Cytosine arabinoside (AraC) [7] and Eribulin [8] are anti-cancer agents derived from sponge-derived natural products. Sorbicillactone-A, which is derived from *Penicillium chrysogenum* (associated with the marine sponge formerly known as *Ircinia fasciculata*, currently accepted as *Sarcotragus fasciculatus*), has also exhibited promising activities against the cytopathic effects of HIV-1 [9]. In addition, sponge-derived microorganisms belonging to at least 35 bacterial and 12 fungal genera have been identified as producing antimicrobials [10]. These natural products include kocurin (against methicillin resistant *Staphylococcus aureus*) [11], sydonic acid (against *Escherichia coli*) [12], trichoderin A (against *Mycobacterium smegmatis*) [13], and saadamycin (against *Candida albicans*) [14].

Microbial natural products are regarded as important components in the modern drug arsenal and are synthesised by a series of enzymes encoded by certain genes localised in biosynthetic gene clusters (BGCs). Most commonly, BGCs encode polyketide synthases (PKS), non-ribosomal peptide synthetase (NRPS), terpenecyclase, and ribosomally synthesised post-translationally modified peptides (RiPPs) [15]. Among these, PKS and NRPS are responsible for synthesising polyketides (PKs) and non-ribosomal peptides (NRPs), respectively. To date, more than 23,000 natural PK and NRP products have been identified and characterised, including antibiotics and agents with anti-tumour, anthelminthic, immunosuppressant, and other biological activities [16]. Thus, the search for genes that encode PKS and NRPS has proven useful for uncovering the biosynthesis potential of a specific organism. Screening for PKS and NRPS genes in sponge-associated bacteria could not only help detect new functional gene sequences but could also provide a basis for revealing the symbiotic relationships between sponges and bacteria and may offer insight into the origin of sponge-derived metabolites [17,18].

Culturing sponge-derived microorganisms that produce interesting metabolites has been deemed the most direct method for the large-scale production of bioactive compounds [19,20]. However, owing to their symbiotic relationships and specific environmental settings, most sponge-associated bacteria cannot be cultured in the laboratory, and their metabolic roles remain unknown [21]. The need to culture more sponge-associated bacteria has led to great efforts to improve culture-dependent methods. So far, multiple culturing condition modifications have been made, including adjustments to nutrients, pH, oxygen levels, and incubation temperatures. These improved conditions enable the recoverability of uncultured sponge-derived bacteria [22]. However, a large majority of BGCs in microorganisms are cryptic in conventional laboratory culturing methods but can be activated using other approaches. Genome sequencing and mining have paved the way for exposing potential secondary metabolites [23,24]. In addition, variations in culturing conditions (e.g., supplementing with antibiotics or specific chemicals, co-culturing, and nutrient screening) [25], mutations [26], and synthetic biology [27] can also trigger the expression of specific metabolite-producing pathways.

The sponge genus *Ophlitaspongia* Bowerbank, 1866 belongs to family *Microcionidae* of order *Poecilosclerida*, class *Demospongiae*, belonging to phylum *Porifera*. To our knowledge, the genus *Ophlitaspongia* has been reported in countries bordering the Atlantic Ocean including Portugal, France, Spain, and the British Isles and have not yet been reported from countries with a Pacific Ocean coastline, including China [28,29]. The sponge genus *Ophlitaspongia* may be a promising source of bioactive compounds. The extracts of six marine sponges belonging to genus *Ophlitaspongia* have previously exhibited strong cytotoxic activity, with inhibition of cell proliferation and high detachment rates in neuroblastoma cultures [30]. A genome-specific analysis performed on a *O. papilla* sponge-derived strain *Endozoicomonas* sp. OPT23 revealed the enrichment of gene clusters encoding for unique metabolic features and adaptive genomic signatures, which might facilitate the specific sponge–bacteria interaction [31]. However, to our knowledge, the antimicrobial activities of microorganisms isolated from *Ophlitaspongia* sp. have not been reported. In this study, we performed antimicrobial activity, biosynthetic potential (PKS and NRPS genes), and phylogenetic analyses of culturable bacteria associated with the sponge *Ophlitaspongia* sp. collected from the Yellow Sea, China. 

## 2. Results

### 2.1. Antimicrobial Activity of Sponge-Associated Bacteria

**Figure 1:**
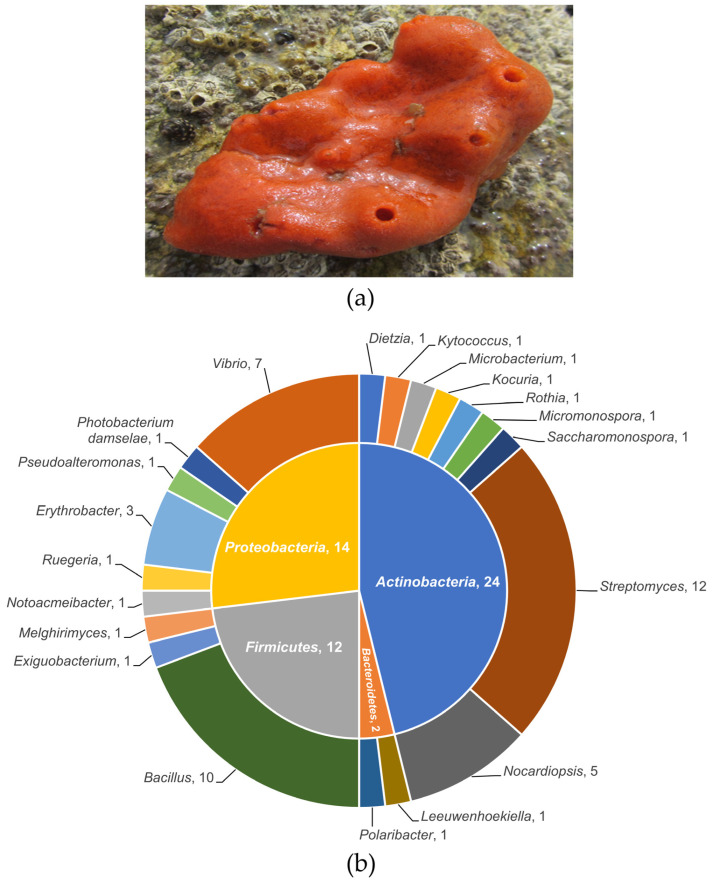
Sponge *Ophlitaspongia* sp. and its associated bacteria. (**a**) Sponge *Ophlitaspongia* sp. sample collected from the Weihai coast of the North Yellow Sea, China. (**b**) A pie chart showing the proportion of the different bacterial genera isolated from the sponge.

A total of 151 sponge-associated bacteria were isolated from *Ophlitaspongia* sp. (Figure 1a). All 151 strains were screened for growth inhibition of seven indicator strains. Of the 151 strains, 94, 36, and 23 exhibited antibacterial activity against at least one, two, and three of the indicator strains, respectively (Table S1). The isolated strains HMA010 (*Dietzia* sp.) and HMA033 (*Streptomyces* sp.) exhibited antimicrobial activities against four indicator strains, while HMA011 (*Streptomyces* sp.) and HMA008 (*Notoacmeibacter* sp.) exhibited antibacterial activities against five of the indicator strains.

The isolated strains exhibited higher antimicrobial activities against Gram-positive bacteria (*Bacillus subtilis* and *S. aureus*) than Gram-negative bacteria (*E. coli*, *Pseudomonas aeruginosa*, *Vibrio parahaemolyticus*, and *V. anguillarum*), with 92 strains (60.93%) inhibiting Gram-positive bacteria, while only 8 strains (5.29%) inhibited Gram-negative bacteria. A total of 83 strains (57%) exhibited inhibitory activity against *S. aureus*, and 19 strains had inhibition zones larger than 12 mm. The isolated strain HMB057, which had a 99.7% similarity with *Ruegeria atlantica* strain CECT 4292, had the strongest inhibitory effects against *S. aureus*, with an inhibition zone diameter of 21.5 mm. A total of 60 strains inhibited *B. subtilis*, of which 10 had inhibition zones larger than 12 mm. The isolated strain HMA033, which was 100% similar to *Streptomyces gougerotii* NBRC 3198, was active against *B. subtilis*, with an inhibition zone of 15.1 mm. HMA033 also exhibited strong inhibitory effects against *C*. *albicans*, with an inhibition zone of 13.8 mm. HMA008 and HMB046 exhibited antimicrobial activities against *P. aeruginosa* and *E. coli*, respectively. In addition, two and six strains exhibited activities against *V. anguillarum* and *V. parahaemolyticus*, respectively. Twenty-eight strains (18.5%) exhibited antimicrobial activities against *C. albicans*, with strain HMA020 displaying the largest inhibition zone (17.5 mm). Strain HMA008 exhibited potent antimicrobial activities against *E. coli*, *P. aeruginosa*, *S. aureus*, *V. anguillarum*, and *V. parahemolyticus*, with inhibition zones >20 mm. 

### 2.2. Phylogenetics of Sponge-Associated Bacteria

Based on their colonial and cellular morphologies, 52 strains were selected for molecular identification and phylogenetic analysis. The 16S rRNA gene-sequencing-based analyses indicate that these strains belonged to 20 genera from 18 families in four phyla, with similarities to type strains in the range of 98.77–100% (Figure 1b and S1). Nearly half (24 strains) belonged to the phylum *Actinobacteria*. They were distributed among nine genera (*Dietzia*, *Kytococcus*, *Microbacterium*, *Kocuria*, *Rothia*, *Micromonospora*, *Saccharomonospora*, *Streptomyces*, and *Nocardiopsis*) from eight families (*Dietziaceae*, *Kytococcaceae*, *Microbacteriaceae*, *Micrococcaceae*, *Micromonosporaceae*, *Pseudonocardiaceae*, *Streptomycetaceae*, and *Nocardiopsaceae*), with *Streptomyces* (12 strains) as the dominant genus. An additional 12 strains were from the phylum *Firmicutes*. These belonged to three genera (*Bacillus*, *Exiguobacterium*, and *Melghirimyces*) in three families (*Bacillaceae*, *Exiguobacteriaceae*, and *Thermoactinomycetaceae*), and most were *Bacillus* (10 strains). In addition, 14 strains were from the phylum *Proteobacteria*, with 5 strains from *Alphaproteobacteria* and 9 from *Gammaproteobacteria*. The *Alphaproteobacteria* strains were distributed among three genera (*Notoacmeibacter*, *Ruegeria*, and *Erythrobacter*) in three families (*Notoacmeibacteraceae*, *Rhodobacteraceae*, and *Erythrobacteraceae*), while the *Gammaproteobacteria* strains were distributed among three genera (*Pseudoalteromonas*, *Photobacterium*, and *Vibrio*) in three families (*Pseudoalteromonadaceae*, *Photobacterium*, and *Vibrionaceae*). *Vibrio* was the dominant *Proteobacteria* genus in this study. Finally, the phylum *Bacteroidetes* was represented in two strains, which were related to the genera *Leeuwenhoekiella* and *Polaribacter* of the family *Flavobacteriaceae*. 

### 2.3. Phylogenetic Analyses of PKS and NRPS Encoding Genes

**Figure 2:**
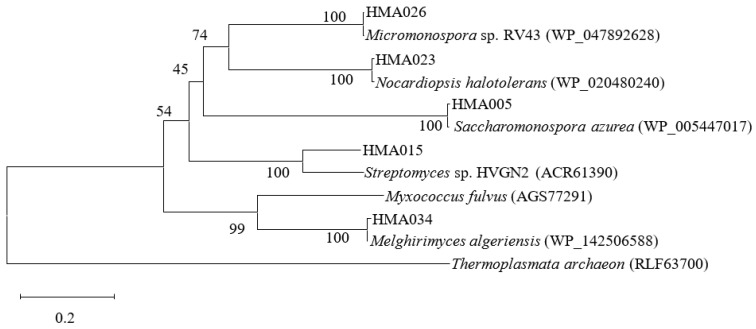
Neighbour-joining phylogenetic tree based on comparisons of the amino acid sequences of the PKS genes of bacterial strains associated with the sponge *Ophlitaspongia* sp. Bootstrap values (>40%) based on 1000 replicates are shown at branch nodes. Bar represents 0.2 amino acid substitution per site.

**Figure 3:**
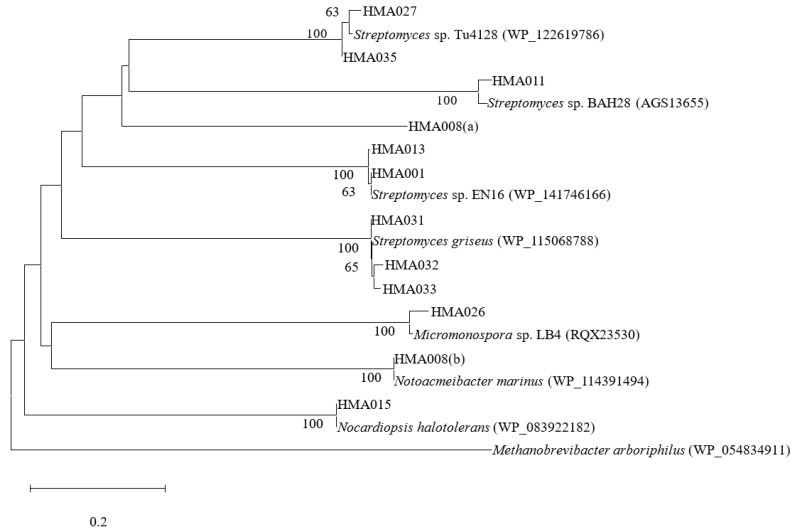
Neighbour-joining phylogenetic tree based on comparisons of the amino acid sequences of the NRPS genes of bacterial strains associated with the sponge *Ophlitaspongia* sp. Bootstrap values (>50%) based on 1000 replicates are shown at branch nodes. Bar represents 0.2 amino acid substitution per site.

Of the 52 strains screened, 13 were positive for PKS or NRPS genes, indicating that these strains possessed at least one BGC encoding for either a polyketide or a non-ribosomal peptide compound (Table 1, Figure 2 and Figure 3). PKS genes were detected in five of the thirteen strains. The translated amino acid sequences of these five strains exhibited 60.27–99.56% similarity with their closest relatives based on BLASTP results. Eleven strains contained NRPS genes and had 95.51–100% amino acid sequence homology with their closest matches that manifested in the A domain fragments. Strains HMA015 (*Nocardiopsis* sp.) and HMA026 (*Micromonospora* sp.) were positive for both PKS and NRPS sequences and exhibited considerable antimicrobial activity. Of the 13 strains containing PKS or NRPS genes, 12 belonged to the phylum *Actinobacteria* and the dominant genus was *Streptomyces*. Furthermore, strain HMA008 (class *Alphaproteobacteria)* harboured two NRPS gene sequences.

### 2.4. HMA008 Genome Sequence Annotation and Mining for Secondary Metabolite Biosynthetic Gene Clusters

**Figure 4:**
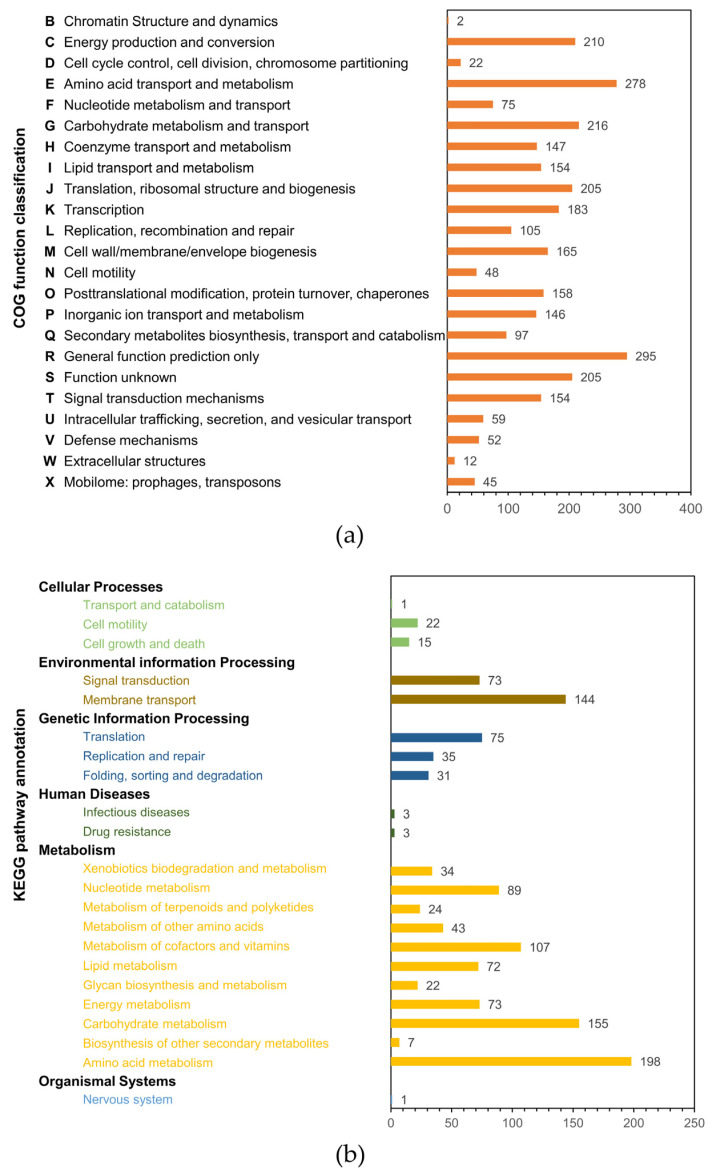
Clusters of Orthologous Groups (COG) and Kyoto Encyclopedia of Genes and Genomes (KEGG) classifications of the HMA008 genome: (**a**) COG analysis; (**b**) KEGG analysis.

Genome sequencing of strain HMA008 (*Notoacmeibacter* sp.) yielded a draft genome that was 3,531,348 bp in length after assembly, producing 22 contigs. The N50 value was 462,813, and the L50 value was 4. The G+C content of strain HMA008 was 61.5%. We investigated the gene functions and metabolic pathways of HMA008 using the Clusters of Orthologous Groups of Proteins (COG) and Kyoto Encyclopaedia of Genes and Genomes (KEGG) databases. A total of 3033 genes were assigned to 23 COG functional categories (Figure 4a). Among these, “general function prediction only” occupied the largest proportion (295 genes), followed by “amino acid transport and metabolism” (278 genes), “carbohydrate transport and metabolism” (216 genes), and “energy production and conversion” (210 genes). Ninety-seven genes were involved in secondary metabolite biosynthesis, transport, and catabolism. Of the KEGG annotated genes, 1227 were classified into six categories, including cellular processes, environmental information processing, genetic information processing, human diseases, metabolism, and organismal systems (Figure 4b). Among these, genes involved in amino acid metabolism (198 genes), carbohydrate metabolism (155 genes), and membrane transport (144 genes) were the most enriched classes. Some pathways were related to terpenoid and polyketide metabolism (24 genes) and the biosynthesis of other secondary metabolites (7 genes). Antibiotics Secondary Metabolites Analysis Shell (antiSMASH) analysis of the contigs detected 12 BGCs putatively encoding for diverse secondary metabolites, including clusters of β-lactone-containing protease inhibitor, butyrolactone, homoserine lactone, N-acetylglutaminylglutamine amide, NRPS, prodigiosin, siderophore, terpene, and other unspecified RiPPs (Table S2). Specifically, two NRPS clusters were present. One of the NRPS clusters exhibited a weak similarity (6%) to the known aurantimycin A BGC, while the other did not match putative gene clusters in the antiSMASH database. Strain HMA008 contained the siderophore cluster, which exhibited 42% similarity to the homologous gene cluster that produces ochrobactin. The β-lactone-containing protease inhibitor gene cluster in HMA008 had a 13% similarity to a homologous gene cluster that produces fengycin. Another BGC of HMA008 exhibited 38% similarity to a homologous gene cluster that produces prodigiosin.

## 3. Discussion

Sponges harbour highly diverse symbiotic microorganism communities and are considered to be one of the major contributors to marine microbial diversity [2]. Although culture-independent techniques, including DNA fingerprinting approaches, metagenomic libraries, and next-generation sequencing techniques, have determined that sponge microbial communities are dominated by >60 phyla, most of the sponge-associated microorganisms cannot be cultured without the host; thus, sponge resources remain untapped [22,32,33,34]. By applying a culture-dependent approach, we successfully isolated 151 strains from the sponge *Ophlitaspongia* sp., as well as their representative strains, which are distributed across 20 genera from 18 families in 4 phyla and include *Actinobacteria*, *Bacteroidetes*, *Firmicutes*, and *Proteobacteria*. Phylogenetic analyses confirmed that all 20 genera of the isolated strains overlapped with previously reported isolates associated with sponges and other marine hosts, such as corals, ascidians, and algae, from different geographical locations obtained using culture-dependent methods [35,36,37,38,39,40,41,42,43,44,45,46]. Genera such as *Bacillus*, *Microbacterium*, *Nocardiopsis*, *Pseudoalteromonas*, *Saccharomonospora*, and *Streptomyces* are distributed throughout aquatic environments and are well-known secondary metabolite producers that may protect host sponges from microbial infection and predators [47,48,49,50,51,52]. For example, at least 740 bioactive substances have been isolated from the genus *Micromonospora*, which produces the largest number of natural products among rare actinomycetes [53]. Chemically diverse products such as new alkaloids and α-pyrones have recently been isolated from *Saccharomonospora* [54,55]. Genera such as *Dietzia*, *Rothia*, *Leeuwenhoekiella*, and *Polaribacter* have served as specific degraders of chemically diverse carbohydrates and may play crucial roles in the marine food web and energy metabolism [56,57,58,59]. Specifically, the genus *Dietzia* is known for degrading hydrocarbons such as polycyclic aromatic compounds [56]. Members of the genus *Rothia* have diverse metabolic capacities, including carbohydrate and peptide fermentation, to primary metabolites, while *Leeuwenhoekiella* species have been found to hydrolyse esters, glucose, and amides, as well as utilise labile carbon sources [60,61]. These genera could serve as helper bacteria that provide growth substrates for specialised bacterial communities [60]. Pigmented strains of the genus *Pseudoalteromonas*, which possessed secondary bioactive metabolites such as bromoalterochromides and indolmycin, also harbour chitinolytic machinery that degrades chitin [62]. Novel species discovered in sponges continue to be added to *Bacillus, Erythrobacter*, *Leeuwenhoekiella*, *Micromonospora*, and *Streptomyces* [37,43,63,64,65]. Previously, Alex et al. [66] used classic isolation techniques with a marine agar 2216 medium (containing Amphotericin B) and observed a high incidence of *Vibrio* sp. (~58%) among the cultured bacterial isolates associated with *O. papilla* and two other sponge hosts collected from the Atlantic coast of Portugal. Moreover, high *Vibrio* sp. abundances were also reported among other host sponges, as *Vibrio* sp. are considered to play an important role in nitrogen fixation [67,68]. However, only a small proportion of the representative microbial community were grouped into *Vibrionales* in this study, and some of the cultured isolates belonged to actinomycetes instead. This difference can be attributed to many factors, including pre-treatment techniques, antibiotic supplements, and culturing media selection. Specifically, in addition to using 2216E, we used four media such as M1 and ISP-2, which have been deemed successful media for the isolation of actinomycetes associated with sponge samples [69]. Dilution samples were also heated prior to inoculation, which could have stimulated the germination of actinomycete spores and inhibited the growth of irrelative genera; however, some *Vibrio* sp. are susceptible to heat, and mild heat treatments might not be effective against their growth [69,70]. 

Antibiotic resistance poses a major ongoing threat to global health security. Therefore, it is crucial to develop novel antimicrobial agents with superior properties and specificity. The antimicrobial assays performed in this study detected a large number of bacterial extracts that were active against three of the indicator strains (*S. aureus*, *B.* *subtilis*, and *C. albicans*). The strains with antimicrobial activities against these three indicator strains were mainly *Actinobacteria* (genera *Dietzia*, *Microbacterium*, *Rothia*, and *Streptomyces*) and *Proteobacteria* (genera *Ruegeria* and *Vibrio*). These two phyla have been identified as the most dominant sponge-derived groups that produce bioactive metabolites [71]. Most active strains inhibited Gram-positive indicator bacteria, while fewer strains exhibited antimicrobial activity against Gram-negative bacteria. Only two strains (HMA008 and HMB04) were observed to inhibit *E. coli.* Similar results have been obtained previously in other sponge species, as well as in other marine invertebrates such as corals and ascidians [44,72]. It is generally considered that the existence of an outer membrane barrier and multi-drug efflux pumps make Gram-negative bacteria more resistant to antibiotics than Gram-positive bacteria [73]. However, the isolate Xp 4.2 associated with the sponge *Xestospongia testudinaria* exhibited a more powerful inhibitory activity against Gram-negative bacteria *(E. coli* and *Klebsiella pneumoniae*) than against Gram-positive bacteria (*Bacillus subtilis*) and displayed the most potent bioactive activity against *E. coli* [74]. 

Detection of genes that encode PKS and NRPS has generally been used to assess the biosynthetic potential of each microbial strain. Among the 52 strains sequenced for functional genes, 14 contained PKS and/or NRPS genes and exhibited antimicrobial activities. Most of these strains were *Actinobacteria*, particularly from the genera *Streptomyces*, which has exhibited extraordinary genetic potential to produce various secondary metabolites [75,76]. Moreover, the *Actinobacteria Micromonospora* sp. HMA026 and *Nocardiopsis* sp. HMA015 had both PKS and NRPS genes. The PKS gene sequence from HMA005 exhibited the closest amino acid similarity (99.11%) with sequences from *Saccharomonospora azurea*, which produces primycin [77]. The NRPS gene sequences of HMA035 and HMA027 exhibited 96.2% and 97.5% similarities at the amino acid level, respectively, with that of *Streptomyces* sp. Tu4128, which produces bagremycins [78]. Similarly, the genes from strains HMA031, HMA032, and HMA033 exhibited the closest BLASTP matches (96–98% amino acid similarities) and were grouped with sequences involved in the production of polycyclic tetramate macrolactams (PTMs), which have been found in bacteria such as *Streptomyces* species and possess antifungal, antibiotic, and antioxidant properties [79]. The remaining 38 strains were negative for PKS and/or NRPS genes, while 26 possessed a broad spectrum of antimicrobial activities. For example, the actinobacterial strain HMA010 (*Dietzia* sp.), which was negative for PKS and NRPS genes, exhibited inhibition against four indicator strains (*B. subtilis*, *S. aureus*, *V. parahemolyticus*, and *C. albicans*). Previous studies have shown that microorganisms have multiple and complex BGCs involved in the production of bioactive products [80,81]. Therefore, these strains with considerable antimicrobial activities may contain other known or potentially novel BGCs responsible for antibiotic biosynthesis.

Genus *Notoacmeibacter*, which belongs to the family *Notoacmeibacteraceae* in the class *Alphaproteobacteria*, was first reported by Huang et al. [82]. To date, *Notoacmeibacter* only comprises two species, *N. marinus* and *N. ruber*, which were isolated from the gut of a limpet and from a *Rhizophorastylosa* leaf, respectively [82,83]. The HMA008 strain was identified as *N. marinus* (99.93% similarity) according to the phylogenetic analysis of 16S rRNA gene sequences. To our knowledge, this is the first time that a strain of the rarely recovered genus *Notoacmeibacter* has been isolated from a marine sponge. We also tested the antimicrobial activity of *Notoacmeibacter* sp. HMA008 for the first time. The results indicate that HMA008 exhibited potent antimicrobial activities against five indicator strains, including both Gram-positive and Gram-negative bacteria. 

The entire HMA008 genome was sequenced and annotated with COG and KEGG. The COG database assesses the phyletic evolution of protein families, while the KEGG database provides a platform to identify the represented metabolic pathways. According to the COG annotation, 6.76% of genes (205) had unknown functions, indicating that there are still many proteins in *N. marinus* that remain to be investigated. An enriched array of the gene sequences was involved in amino acid and carbon hydrate transport and metabolism functions, which might support higher levels of energy production and conversion by HMA008. The potential metabolic activities revealed by KEGG, as well as the membrane transport function, also suggest a complex secondary metabolism for strain HMA008. Secondary metabolite gene cluster analysis revealed 12 BGCs responsible for producing diverse bioactive metabolites, such as β-lactone natural products, NRPs, prodigiosin, and siderophores. One of the NRPS clusters displayed a weak similarity to the known clusters potentially encoding aurantimycin A, a depsipeptide antibiotic with cytotoxic properties. The other NRPS clusters, as well as RiPP-like clusters, did not match the antiSMASH database, which may due to the incomplete genome assemblies acquired by next-generation sequencing technologies. Siderophores are known to act effectively against drug-resistant pathogens such as *S. aureus*, *P. aeruginosa*, and *A. baumannii* [84]. β-lactone natural products have drawn significant attention for their potent bioactivity against bacteria, fungi, and human cancer cells [85]. Prodigiosin has also been described as a bacteriostatic agent on *E. coli* [86,87]. Collectively, the genome mining of the BGCs in HMA008 indicates that the strain produces many secondary metabolites that might make it capable of inhibiting a broad spectrum of indicator strains, including *S. aureus*, *Escherichia coli*, *P. aeruginosa*, *V. anguillarum*, and *V. parahemolyticus*. Our results provide preliminary evidence for the antimicrobial potential of *N. marinus*. Further studies are necessary to identify the bioactive substances produced by HMA008.

Furthermore, strain HMA034, which belongs to the phylum *Firmicutes*, is closely related to *Melghirimyces algeriensis* NariEX (99.65% similarity) of the family *Thermoactinomycetaceae*. At least 43 species belonging to 21 genera of the family *Thermoactinomycetaceae* have been discovered to date in a variety of natural environments, including hot springs, sediment, and the human gut [88,89,90]. Recent research on microorganisms associated with the sponge *Scopalina hapalia* observed that an unidentified genus related to the family *Thermoactinomycetaceae* had tyrosinase inhibitory activities, which indicated its potential to overcome skin aging [91]. In this study, we found potential antimicrobial activities of the *Thermoactinomycetaceae* species against *B. subtilis*, *S. aureus*, and *C. albicans*. In addition, biosynthetic potential mining indicates that it contains the PKS gene. Thus, the *Thermoactinomycetaceae* strains may represent a promising source for discovering new antimicrobial agents. 

In conclusion, the results presented in this study suggest that the sponge *Ophlitaspongia* sp. from the Yellow Sea harbours diverse culturable bacteria. Many of the strains had antimicrobial activities, and a number of them were positive for the amplification of PKS and NRPS genes, suggesting that these sponge-derived strains may be promising sources of bioactive secondary metabolites. In addition, strain *Notoacmeibacter* sp. HMA008 had potent antimicrobial activities and several BGCs that encode secondary metabolites. Further studies are needed that focus on isolating and identifying the potential bioactive compounds produced by these strains.

## 4. Materials and Methods

### 4.1. Sample Collection and Preparation

*Ophlitaspongia* sp. (Figure 1a) samples (n = 10 specimens) were collected from the intertidal zone of Xiaoshi Island (37°31′49′′ N, 122°0′5′′ E) off the Weihai coast in the North Yellow Sea, China, in June 2017. After collection, fresh samples were immediately placed into sterile plastic containers in an icebox and transported to the laboratory within 1–2 h. The sponge samples were rinsed three times with filter-sterilised natural seawater to remove loosely attached microorganisms and other foreign bodies attached to the sponge surfaces. Following this, three sponge samples were cut with sterile tweezers and scissors into pieces of approximately 0.5 cm^3^, which were then ground into homogenates with filter-sterilised natural seawater.

### 4.2. Isolation of Culturable Bacteria

The sample homogenates were added to sterile flasks with 10 volumes of filter-sterilised natural seawater and glass beads, shaken on a rotary shaker (180 rpm) for 30 min, and mixed thoroughly. Following this, the supernatant was diluted across a 10-fold concentration series in filter-sterilised natural seawater ranging from 10^−2^ to 10^−5^ and then subsequently plated on Petri dishes containing one of the five kinds of culture media. The media used were 2216E, M1, Glucose-Yeast-Peptone (GPY), International Streptomyces Project Medium No. 2 (ISP-2, also referred to as Yeast Extract-Malt Extract), and Reasoner’s 2A (R2A) [92]. All of the media were prepared with 1.8% (*w/v*) agar. Inoculated plates were incubated at 28 °C for 7–30 d and were monitored daily throughout the cultivation period.

To obtain greater numbers of Actinobacteria, the sample homogenates were incubated in a 55 °C water bath for 10 min. The supernatant was then serially diluted and plated on Petri dishes containing one of the five kinds of culture media (2216E, M1, GPY, ISP2, and R2A). All of the media were prepared with 1.8% (*w/v*) agar and were supplemented to 20 μg mL^−1^ nalidixic acid and 100 μg mL^−1^ cycloheximide final concentrations, which were used to inhibit the growth of Gram-negative bacteria and fungi [90], respectively. The inoculated plates were incubated at 28 °C for at least 3 weeks and were monitored daily throughout the cultivation period.

### 4.3. Selection and Preservation of Collected Strains

Single colonies growing on the media plates were selected based on colony phenotypical characteristics, including growth rate, morphology, size, pigmentation, and margin characteristics. Following subculture and confirmation of strain purity, the collected bacteria were preserved in 15% glycerin (*v/v*) at −80 °C for further study.

### 4.4. Preparation of Crude Extracts and Antibacterial Activity Screening

All isolated bacteria (151 strains) were analysed for their antimicrobial activities against seven indicator strains. The indicator strains included *Escherichia coli* (ATCC 25922), *Pseudomonas aeruginosa* (ATCC 27853), *Bacillus subtilis* (ATCC 6633), *Staphylococcus aureus* (ATCC 6538), *Candida albicans* (ATCC 10231), *Vibrio parahaemolyticus* (ATCC 17802), and *Vibrio anguillarum*. Most of the indicator strains were purchased from the ATCC, except for *V*. *anguillarum*, which was donated by Associate Professor Yu-Xia Zou from the Institute of Oceanology, Chinese Academy of Sciences. 

Prior to antimicrobial activity assays, the seven indicator strains were inoculated in Mueller–Hinton (MH) liquid medium (beef extract 3 g; soluble starch 1.5 g; acid hydrolysate of casein 17.5 g; distilled water 1 L; pH 7.3 ± 0.2) overnight at 37 °C and 100 µL of each indicator strain suspension adjusted to 0.5 McFarland standard was plated on MH agar (MH with 18 g of agar).

Antimicrobial activity was determined by observing the growth inhibition of bacteria or fungi according to previous protocols [93]. The test strains were inoculated into 50 mL of liquid medium (2216E medium for bacteria, M1 medium for Actinobacteria) and incubated at 28 °C for approximately 5–7 d (bacteria) and 7–10 d (Actinobacteria) on a rotary shaker. The culture supernatants were subsequently extracted three times with 50 mL ethyl acetate. The organic layers were separated, combined, dried over anhydrous sodium sulphate, decanted, and dried under vacuum to obtain a crude extract. The crude extracts of each of the strains were dissolved in dimethyl sulfoxide (DMSO) at a concentration of 25 mg mL^−1^, after which 10 μL of the crude extract solution was added to 6 mm diameter filter papers. The filter paper disks were then placed onto the surface of the MH agar plates with the indicator strains. The same amount of DMSO was used as a negative control, and crude extracts of Marine 2216E or M1 media were also used as negative controls. Chloramphenicol (30 μg), gentamicin (10 μg), and nystatin (10 μg) (Sigma-Aldrich, St Louis, MO, USA) were used as positive controls. The antimicrobial assay plates were then incubated at 37 °C for 24 h for the bacterial indicator strains, and at 37 °C for 48 h for *C. albicans.* The inhibition zones around the paper disks were measured to gauge the antimicrobial activities of the collected strains. Three or more replicates were performed for each isolate to establish average inhibition zone sizes. The inhibition degrees against indicator strains used the following symbols: (−), no inhibition; (+), 6 < inhibition zone < 8 mm; (++), 8 ≤ inhibition zone < 10 mm; (+++), 10 ≤ inhibition zone < 12 mm; (++++), inhibition zone ≥ 12 mm. The isolated strain for which each inhibition zone size was greater than 12 mm exhibited higher antimicrobial activities. 

### 4.5. 16S rRNA Gene Sequencing and Phylogenetic Analyses

Fifty-two strains were selected based on their colony morphologies on Marine 2216E solid medium and the results of Gram staining, after which their 16S rRNA genes were sequenced to determine their phylogenetic positions. Bacteria were cultured in the 2216E liquid medium and Actinobacteria were cultured in the M1 liquid medium. Extraction of genomic DNA and PCR amplification were performed as described by a previous study [46]. Cells were harvested at stationary-phase by centrifugation (10,000× *g* for 1 min). Genomic DNA was extracted by using a Bacterial Genome DNA Extraction Kit (Sangon Biotech, Shanghai, China). The universal primers 27F (5’-AGAGTTTGATCCTGGCTCAG-3’) and 1492R (5’-GGTTACCTTGTTACGACTT-3’) were used in polymerase chain reaction (PCR) amplifcation of the 16S rRNA gene. Extracted genomic DNA was used as a PCR template. Genomic DNA from the bacterial strain *E*. *coli* was used as a positive control, and sterile distilled water was used as a negative control. DNA was denatured at 94 °C for 5 min, followed by 35 cycles of 94 °C for 1 min, 56 °C for 1 min, and 72 °C for 90 s, with a final 10 min extension at 72 °C. PCR products (1500 bp) were sequenced by Sangon Biotech, Shanghai, China. 

Sequence identity searches and calculations of pairwise similarity values between collected strains and their closely related types were performed using the EzBioCloud Database [94]. 16S rRNA gene sequences of the related strains were downloaded from the National Center for Biotechnology Information (NCBI) GenBank database (http://www.ncbi.nlm.nih.gov (accessed on 10 May 2022). Sequences were further analysed by performing multiple sequence alignments using Clustal X [95]. A phylogenetic tree was constructed using the neighbour-joining algorithm, which was implemented using the MEGA (v.6.0) software package [96]. Phylogenetic analyses used *Pyrococcus abyssi* GE5^T^ (L19921) as the outgroup. The 16S rRNA gene sequences of the cultured bacteria were submitted to the NCBI GenBank Database under Accession Numbers MG905366–MG905417.

### 4.6. Genome Sequencing and Secondary Metabolite Cluster Analysis

Genome sequencing of strain HMA008 was performed on an Illumina HiSeq PE150 platform by Novogene Bioinformatics Technology Co., Ltd. (Beijing, China). A-tailed, ligated to paired-end adaptors, and PCR amplified with a 350 bp insert was used for library construction. SOAP denovo (v.2.04) (SOAP, RRID:SCR_000689) was used to assemble genome sequences. The genome sequences were submitted to the NCBI GenBank Database under accession number SAMN09273368 and assembly accession number ASM333676v1. Functional annotation of genes was predicted using COG (http://www.ncbi.nlm.nih.gov/COG/ (accessed on 26 July 2022) [97] and KEGG (http://www.genome.jp/kegg/ (accessed on 26 July 2022) [98]. Secondary metabolite BGCs were predicted using antiSMASH (https://antismash.secondarymetabolites.org (accessed on 26 July 2022) [99]. 

### 4.7. Screening for PKS and NRPS Genes

The presence of genes (PKS and NRPS) involved in the production of secondary metabolites was screened from 52 strains that had undergone 16S rRNA gene sequencing. Genomic DNA was extracted as described above. DKF (5’GTGCCGGGTNCCRTGNGYYTC3’) and DKR (5’GCGATGGAYCCNCARCARYG3’) primers were used for PCR amplification of the gene clusters encoding the KS domain using the extracted genomic DNA as a template [100]. NRPS gene clusters that encode the adenylation (A) domain were amplified using A3F (5′GCSTACSYSATSTACACSTCSGG3′) and A7R (5′SASGTCVCCSGTSCGGTAS3′) primers [101]. DNA from the previously characterised strain *Bacillus* sp. HSA006, which contains PKS and NRPS systems, was used as the positive control and sterile distilled water was used as the negative control [102]. PCR cycles comprised an initial denaturation step of 5 min at 95 °C, 35 cycles of 30 s at 95 °C, 2 min at 57 °C, and 4 min at 72 °C, with a final extension of 10 min at 72 °C. PCR amplification products, corresponding to 650–700 bp for the KS domain of PKS and 700–800 bp for the A domain of NRPS, were purified using the SanPrep Column DNA Gel Extraction Kit (Sangon Biotech, Shanghai, China). The PCR products were ligated into pMD18-T (Takara Bio. Inc., Dalian, China). M13 universal primers were used for sequencing, which was performed by Sangon Biotech. Translated protein sequences were derived from the nucleotide sequences using the Open Reading Frame (ORF) Finder search program available on the NCBI website (https://www.ncbi.nlm.nih.gov/orffinder/ (accessed on 5 February 2022). The deduced amino acid sequences were used as queries to search for related proteins in the Non-redundant (NR) Protein Database using the BLASTP algorithm. Multiple sequence alignments of nucleotide sequences were performed using Clustal X (RRID:SCR_017055), and phylogenetic trees based on the amino acid sequences encoded by the PKS or NRPS genes were constructed using the neighbour-joining method in MEGA (v6.0) (MEGA Software, RRID:SCR_000667) combined with bootstrap analysis using 1000 replications. The phylogenetic analyses used *Thermoplasmata archaeon* (RLF63700) and *Methanobrevibacter arboriphilus* (WP_054834911) as outgroups, respectively. The PKS and NRPS gene sequences were submitted to the NCBI GenBank Database under Accession Numbers MG993566−MG993571, MH024360−MH024364, MH045857−MH045858, and MH050930−MH9050932. 

## Figures and Tables

**Table 1:** Comparison of 16S rRNA genes and amino acid sequences of *polyketide synthase (PKS) and non-ribosomal peptide synthetase (NRPS)* genes of bacterial strains associated with the sponge *Ophlitaspongia* sp.

Test Strain	16S rRNA Gene Homology (%) with Type Strains	PKS Amino Acid Sequence Homology (%)	NRPS Amino Acid Sequence Homology (%)
HMA001	*Streptomyces pratensis* NRRLB 24916^T/^JQ806215/99.88	-	*Streptomyces* sp. EN16/WP_141746166/100
HMA005	*Saccharomonospora azurea* DSM 44631^T/^Z38017/99.71	*Saccharomonospora azurea*/WP_005447017/99.11	-
HMA008	*Notoacmeibacter marinus* MCCC 1A01882^T/^NR157752/99.93	-	NRPS a: *Notoacmeibacter marinus*/WP_114391483/100
			NRPS b: *Notoacmeibacter marinus*/WP_114391494/100
HMA011	*Streptomyces iakyrus* DSM 40482^T/^NR041231/100	-	*Streptomyces* sp. BAH28/AGS13655/95.51
HMA013	*Streptomyces griseolus* NBRC 3415^T/^NR041207/99.87	-	*Streptomyces* sp. EN16 WP_141746166/98.72
HMA015	*Nocardiopsis halotolerans* DSM 44410^T/^AJ290448/100	*Streptomyces* sp. HVGN2/ACR61390/76.92	*Nocardiopsis halotolerans*/WP_083922182/100
HMA023	*Nocardiopsis halotolerans* DSM 44410^T/^AJ290448/99.71	*Nocardiopsis halotolerans* DSM4410/WP_020480240/99	-
HMA026	*Micromonospora aurantiaca* ATCC 27029^T/^NR074415/100	*Micromonospora* sp. RV43/WP_047892628/99.56	*Micromonospora* sp. LB4/RQX23530/96.52
HMA027	*Streptomyces malachitospinus* NBRC 101004^T/^AB249954/99.86	-	*Streptomyces* sp. Tu4128/WP_122619786/97.5
HMA031	*Streptomyces gougerotii* NBRC 3198^T/^NR041201/100	-	*Streptomyces griseus*/WP_115068788/98.25
HMA032	*Streptomyces gougerotii* NBRC 3198^T/^NR041201/100	-	*Streptomyces griseus*/WP_115068788/98.69
HMA033	*Streptomyces gougerotii* NBRC 3198^T/^NR041201/100	-	*Streptomyces griseus*/SUP57408/98.69
HMA034	*Melghirimyces algeriensis* DSM 45474^T/^HQ383683/99.65	*Melghirimyces algeriensis*/WP_142506588/99.55	-
HMA035	*Streptomyces malachitospinus* NBRC 101004^T^/AB249954/99.87	-	*Streptomyces* sp. Tu4128/WP_122619786/96.2

“-” indicates the absence of amplicons for the targeted biosynthetic gene cluster (PKS or NRPS) being evaluated.

## Data Availability

The data used to support the findings of this study are included within the article. The genome sequences were submitted to the NCBI GenBank Database under accession number SAMN09273368 and assembly accession number ASM333676v1.

## Supplementary Materials

The following supporting information can be downloaded at: https://www.mdpi.com/article/10.3390/md20100588/s1, Table S1: Antimicrobial activities of the isolated strains associated with the sponge *Ophlitaspongia* sp.; Table S2: Secondary metabolite biosynthetic gene clusters (BGCs) in the genome of *Notoacmeibacter* sp. HMA008; Figure S1: Neighbour-joining phylogenetic tree based on comparisons of the 16S rRNA sequences of bacterial strains associated with the sponge *Ophlitaspongia* sp. and other related taxa.
