# Supplementary material for: Antimicrobial Biosynthetic Potential and Phylogenetic Analysis of Culturable Bacteria Associated with the Sponge Ophlitaspongia sp. from the Yellow Sea, China
Source: Mar Drugs. 2022 Sep 21;20(10):588. doi: 10.3390/md20100588 (PMC9605435; doi:10.3390/md20100588)
Supplement: Supplementary file 1 [file marinedrugs-20-00588-s001.zip › marinedrugs-1868281-supplementary.pdf]

Supplementary Materials

Table S1 Antimicrobial activities of the isolated strains associated with the sponge *Ophlitaspongia* sp.

Test strains	Inhibition degree against indicator strains						
	Bs	Sa	Ec	Pa	Va	Vp	Ca
HMA001	++	++	-	-	-	-	-
HMA003	-	++++	-	-	-	-	-
HMA004	+++	++	-	-	-	-	-
HMA005	+++	++	-	-	-	-	+++
HMA006	++	++	-	-	-	-	-
HMA007	-	++	-	-	-	-	-
HMA008	-	++++	++++	++++	++++	++++	-
HMA010	+++	++++	-	-	-	+	++
HMA011	++++	+++	-	-	++	++	+++
HMA012	++	++	-	-	-	-	++
HMA013	+	+++	-	-	-	-	-
HMA015	+	++	-	-	-	-	++
HMA016	++	+++	-	-	-	-	+++
HMA019	-	+	-	-	-	-	-
HMA020	-	+	-	-	-	-	++++
HMA022	-	++++	-	-	-	-	-
HMA023	+++	+++	-	-	-	++	-
HMA025	-	+++	-	-	-	-	-
HMA026	+++	+++	-	-	-	-	+++
HMA027	+++	++++	-	-	-	-	+++
HMA028	-	+++	-	-	-	-	-
HMA029	+	+	-	-	-	-	-
HMA031	-	++	-	-	-	+	+++
HMA032	++++	-	-	-	-	-	++
HMA033	++++	+	-	-	-	+	++++
HMA034	+++	++++	-	-	-	-	++
HMA035	+++	+++	-	-	-	-	+++
HMB001	++	-	-	-	-	-	-
HMB005	++	++	-	-	-	-	++
HMB008	-	-	-	-	+	++	++
HMB009	+	+++	-	-	-	-	-
HMB010	+	+	-	-	-	-	++
HMB012	+	+++	-	-	-	-	-
HMB014	-	+++	-	-	-	-	-
HMB019	++	++	-	-	-	-	+++
HMB020	+++	++	-	-	-	-	-
HMB021	+++	++	-	-	-	-	-

Table S1 (continued)

Test strains	Inhibition degree against indicator strains						
	Bs	Sa	Ec	Pa	Va	Vp	Ca
HMB022	++	+++	-	-	-	-	-
HMB023	++	++	-	-	-	-	-
HMB024	++	++++	-	-	-	-	-
HMB025	+++	-	-	-	-	-	-
HMB026	-	++	-	-	-	-	-
HMB028	-	++	-	-	-	-	-
HMB030	-	++	-	-	-	-	-
HMB031	+	+++	-	-	-	-	-
HMB032	-	+++	-	-	-	-	++
HMB033	-	++	-	-	-	-	-
HMB037	++	-	-	-	-	-	-
HMB038	-	++	-	-	-	-	-
HMB040	++	++	-	-	-	-	-
HMB041	+	+	-	-	-	-	-
HMB042	-	++++	-	-	-	-	-
HMB046	++++	++++	++	-	-	-	-
HMB047	++	++	-	-	-	-	-
HMB048	-	+	-	-	-	-	-
HMB050	++++	++++	-	-	-	-	++
HMB054	++	+++	-	-	-	-	-
HMB057	-	++++	-	-	-	-	-
HMB058	-	++	-	-	-	-	-
HMB060	+++	+++	-	-	-	-	-
HMB061	++++	-	-	-	-	-	+++
HMB063	++	-	-	-	-	-	-
HMB064	++	++	-	-	-	-	-
HMB065	-	++	-	-	-	-	-
HMB066	-	++	-	-	-	-	-
HMB067	-	++	-	-	-	-	-
HMB069	-	++++	-	-	-	-	-
HMB070	-	++	-	-	-	-	-
HMB072	+++	+++	-	-	-	-	-
HMB080	-	-	-	-	-	-	+++
HMB083	+++	+++	-	-	-	-	++
HMB085	++	-	-	-	-	-	++
HMB087	-	++++	-	-	-	-	-
HMB088	-	+++	-	-	-	-	-
HMB091	++++	++	-	-	-	-	-
HMB092	++	++	-	-	-	-	-

Table S1 (continued)

Test strains	Inhibition degree against indicator strains						
	Bs	Sa	Ec	Pa	Va	Vp	Ca
HMB093	+	+++	-	-	-	-	-
HMB094	++	++	-	-	-	-	-
HMB095	+	+++	-	-	-	-	-
HMB096	++	++++	-	-	-	-	++++
HMB097	-	++++	-	-	-	-	-
HMB098	+++	+++	-	-	-	-	++++
HMB099	-	++	-	-	-	-	-
HMB100	-	++++	-	-	-	-	-
HMB102	++++	++++	-	-	-	-	++
HMB105	-	+++	-	-	-	-	-
HMB106	-	+++	-	-	-	-	-
HMB107	++++	++++	-	-	-	-	-
HMB108	++	-	-	-	-	-	-
HMB109	+++	+++	-	-	-	-	-
HMB110	+++	++++	-	-	-	-	-
HMB111	+	+++	-	-	-	-	-
HMB113	++	-	-	-	-	-	-
HMB114	++++	+++	-	-	-	-	+++

Only the bacterial strains with antimicrobial activities against at least three indicator strains were listed on the table.

Antimicrobial activities were tested against *Bacillus subtilis* (Bs), *Staphylococcus aureus* (Sa), *Escherichia coli* (Ec), *Pseudomonas aeruginosa* (Pa), *Vibrio anguillarum* (Va), *Vibrio parahemolyticus* (Vp), and *Candida albicans* (Ca).

Symbols of inhibition degree against indicator strains: (−), no inhibition; (+), 6 < inhibition zone < 8 mm; (++), 8 ≤ inhibition zone < 10 mm; (+++), 10 ≤ inhibition zone < 12 mm; (++++), inhibition zone ≥ 12 mm.

Table S2 Secondary metabolite biosynthetic gene clusters (BGCs) in the genome of *Notoacmeibacter* sp. HMA008.

Type	Location (From-To)	Similar known cluster	Classification	Similarity (%)	Core gene locus tag
Beta-lactone containing protease inhibitor	189059-212033	Fengycin	NRP	13%	ctg17_184, ctg17_185
Beta-lactone containing protease inhibitor	189509-212033	Fengycin	NRP	13%	ctg27_186, ctg27_187
Butyrolactone cluster	634359-645132	-	-	-	ctg18_588
Butyrolactone cluster	134264-145037	-	-	-	ctg23_117
Homoserine lactone cluster	17559-31574	-	-		ctg8_38
N-acetylglutaminylglutamine amide	489072-503734	-	-	-	ctg15_478, ctg15_479, ctg15_480
NRPS	1-13248	-	-	-	ctg4_9
NRPS	1-13375	Aurantimucin A	NPR+Polyketide	6%	ctg5_7
Prodigiosin	4334-40131	Prodigiosin	Polyketide	38%	ctg9_31, ctg9_35, ctg9_36, ctg9_37, ctg9_41
RiPP-like	206011-216895	-	-	-	ctg15_212
Siderophore cluster	1949-16218	Ochrobactin	Others	42%	ctg14_9, ctg14_11
Terpene	74124-94957	-	-	-	ctg13_82

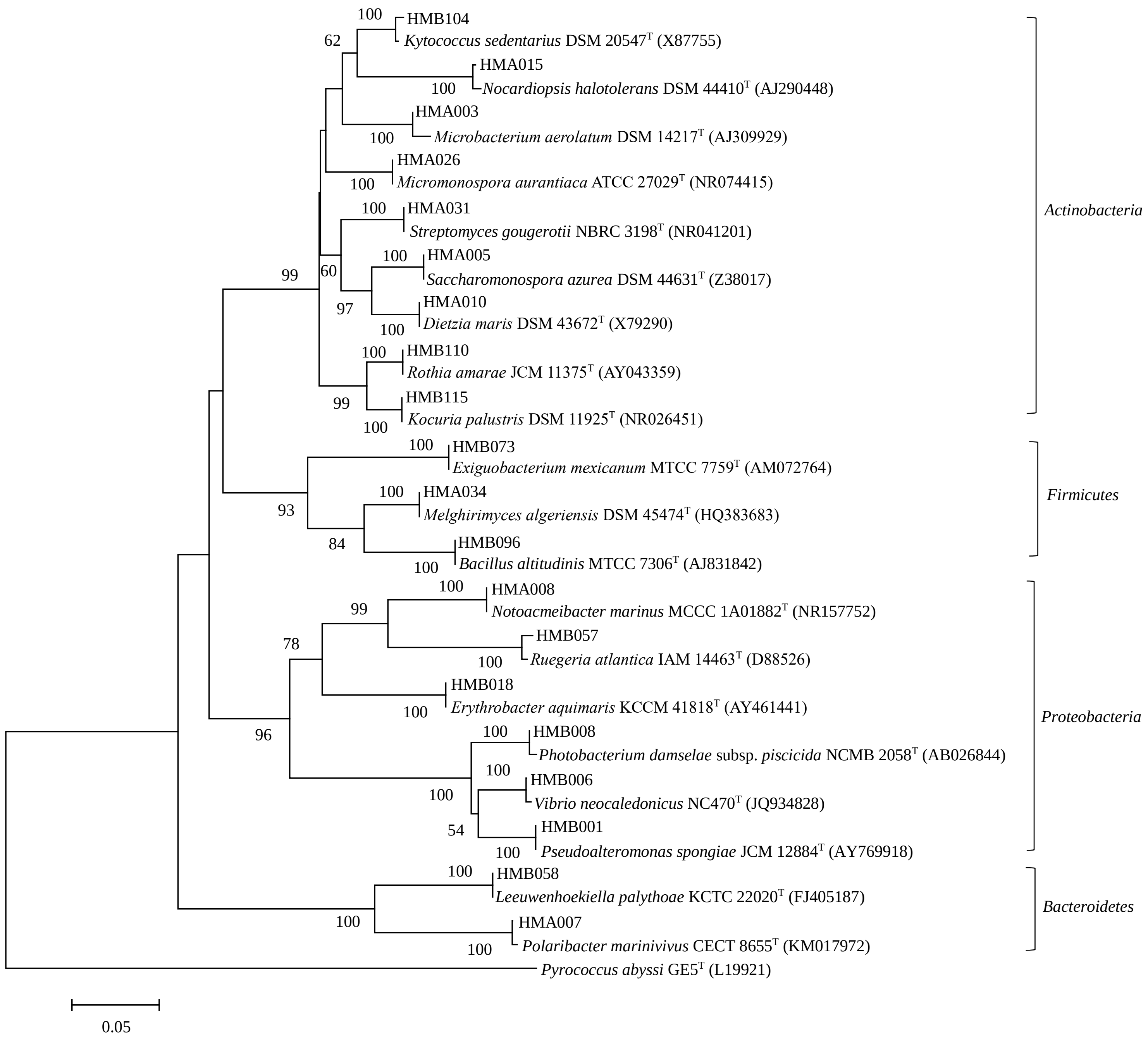

Figure S1 Neighbour-joining phylogenetic tree based on comparisons of the 16S rRNA sequences of bacterial strains associated with the sponge *Ophlitaspongia* sp. and other related taxa. There are a total of 20 genera collected from the sponge, and we choose one species from each genus. The numbers on the tree indicate the percentages of bootstrap based on 1,000 replications and are shown for branches with more than 50% support. Bar represents 0.05 substitutions per nucleotide position.
